# Palatine Tonsillar Infection by *Pseudoterranova azarasi*

**DOI:** 10.4269/ajtmh.20-0175

**Published:** 2020-07-08

**Authors:** Sho Fukui, Takahiro Matsuo, Nobuyoshi Mori

**Affiliations:** 1 Immuno-Rheumatology Center, St. Luke’s International Hospital, Tokyo, Japan;; 2 Department of Infectious Diseases, St. Luke’s International Hospital, Tokyo, Japan

A 25-year-old woman presented with a 5-day history of left pharyngeal pain and irritation after consuming assorted sashimi. Physical examination identified a black moving worm in the left palatine tonsil. Her blood test results were normal. Symptoms rapidly improved after removing the worm using tweezers.

The worm body was black, 38 mm long, 1 mm wide, and was molting the outer cuticle (Figure 1A, Supplemental Video). DNA PCR and the fact that the worm was in exuviation revealed this worm was a fourth-stage larva of *Pseudoterranova azarasi* (Figure 1B).

**Figure 1.**
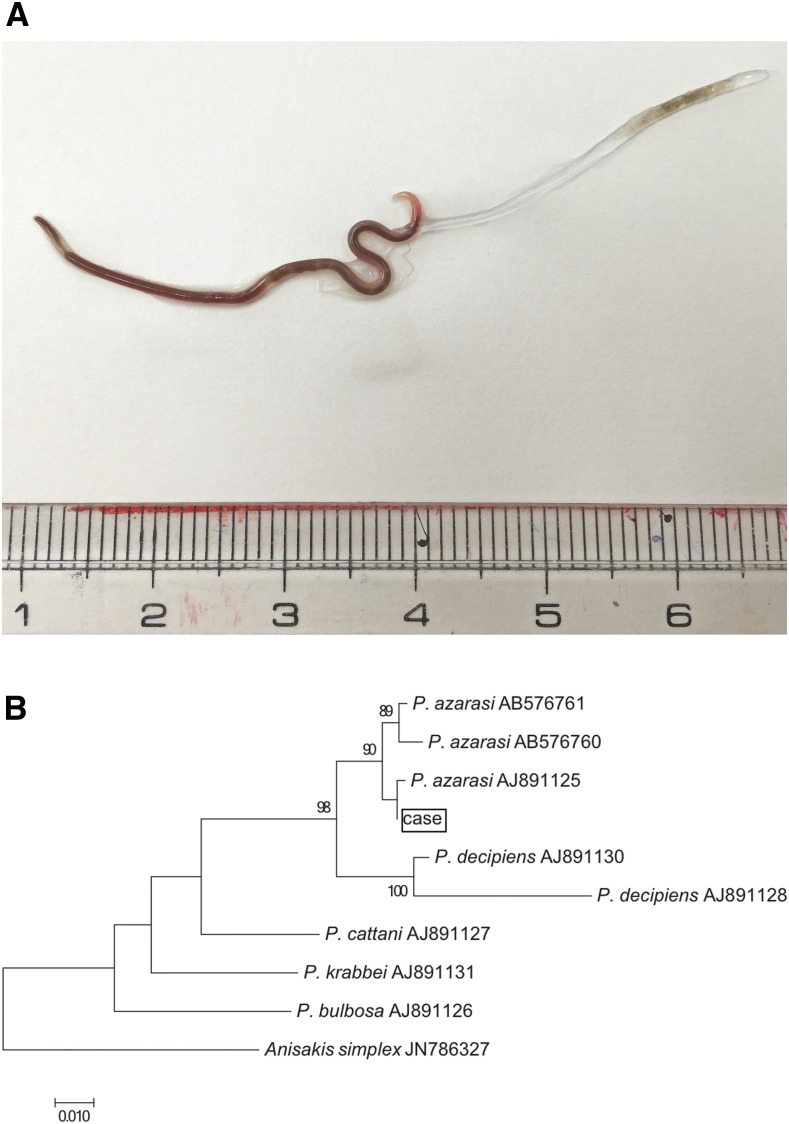
(**A**) The worm body of *Pseudoterranova azarasi.* The worm was molting the outer cuticle. (**B**) The phylogenetic tree of the worm obtained and related sequences based on mitochondrial nicotinamide adenine dinucleotide hydride dehydrogenase subunit 1 (NAD1) sequence (498 positions). This figure appears in color at www.ajtmh.org.

*Pseudoterranova* is an uncommon nematode of the family Anisakidae. *Anisakis simplex*, a major type of the family, is a white roundworm causing gastric, intestinal, ectopic, and allergic diseases.^1^ Just like *Anisakis*, *Pseudoterranova* infects dominantly in the stomach after consuming third-stage larvae in raw or undercooked marine fish, and more than 700 cases have been reported in Japan, North Pacific countries, South America, and the Netherlands.^2–4^

*Pseudoterranova* infection is diagnosed based on clinical course and morphological features because anti–*Anisakis* sp. antibody is insensitive^4^ and PCR is not commercially available. Therefore, clinicians should be aware of differences from *Anisakis* infection. *Pseudoterranova* bodies are larger and darker, and symptoms are milder than in *Anisakis* infection.^1^ There is limited evidence of pharmacological treatment; direct removal is the most effective.

Although oropharyngeal infection is rare, this infection is known to cause “tingling throat syndrome” and cough^5^ and should be considered a differential diagnosis of oropharyngeal parasitosis as consuming raw fish, including sushi and sashimi, has become more popular and the number of reported cases has markedly increased worldwide.

## Supplemental movie

Supplemental materials
